# Supplementary material for: van der Waals forces control ferroelectric–antiferroelectric ordering in CuInP2S6 and CuBiP2Se6 laminar materials
Source: Chem Sci. 2018 Sep 17;9(39):7620–7. doi: 10.1039/c8sc01274a (PMC6187460; doi:10.1039/c8sc01274a)
Supplement: Supplementary file 1 [file SC-009-C8SC01274A-s001.pdf]

Electronic Supplementary Material (ESI) for Chemical Science.
This journal is © The Royal Society of Chemistry 2018

Van der Waals forces control ferroelectric-antiferroelectric ordering in CuInP2S6 and CuBiP2Se6 laminar materials

Jeffrey R. Reimers,[a,b] Sherif Abdulkader Tawfik[b], and Michael J. Ford[b]

[a] International Centre for Quantum and Molecular Structures and School of Physics, Shanghai University, Shanghai 200444, China

[b] School of Mathematical and Physical Sciences, University of Technology Sydney, Ultimo, New South Wales 2007, Australia

email: Jeffrey.Reimers@uts.edu.au, reimers@shu.edu.cn, sherif.abbas@uts.edu.au, Mike.Ford@uts.edu.au

Electronic Supplementary Information

PBE-D3 optimized coordinates

```
CuInP2S6 bilayer F [bc] PBE-D3

E=  -93.7897 eV
box:   6.088000 0 0  -3.044000   5.272363 0 0 0  30.000000
Kpoints:  4  4  1
basis: NGX=  36 NGY=  36 NGZ= 168 NGXF=  72 NGYF=  72 NGZF= 336

 16    2.108660   -1.629990   -1.375360
 16   -2.108620    1.886808    1.843055
 16    1.860359    1.696268   -1.398392
 16    3.985952   -1.876281    1.870854
 16   -0.896467   -0.188876   -1.364746
 16    1.160186    0.016238    1.857509
 16   -0.084554   -1.909312   -4.786738
 16    0.086267    1.614009    5.255572
 16   -0.071032    1.854749   -4.775819
 16   -0.154037   -1.728503    5.248953
 16   -2.903816   -0.040716   -4.778229
 16   -2.929050    0.153250    5.253683
 15    4.064685   -1.793004   -1.996765
 15   -4.058906    1.766839    2.380786
 15    4.055182   -1.789774   -4.252653
 15   -4.044558    1.770443    4.636218
 49    1.009890   -0.038461   -3.344876
 49   -1.006855    0.014689    3.291755
 29   -2.015966    1.714916   -1.617053
 29    2.046517   -1.744146    4.910261
```

```
CuInP2S6 bilayer Aii [a'b] PBE-D3

E=  -93.8832 eV
box:   6.088000 0 0  -3.044000   5.272363 0 0 0  30.000000
Kpoints:  4  4  1
basis: NGX=  36 NGY=  36 NGZ= 168 NGXF=  72 NGYF=  72 NGZF= 336
```

16	-0.882747	-0.186261	-1.615606
16	0.882747	0.186261	1.615606
16	2.122095	-1.629497	-1.602588
16	-2.122095	1.629497	1.602588
16	1.870607	1.694787	-1.624633
16	-1.870607	-1.694787	1.624633
16	-2.891906	-0.046892	-5.011951
16	2.891906	0.046892	5.011951
16	-0.058576	1.849399	-5.016168
16	0.058576	-1.849399	5.016168
16	-0.073869	-1.917399	-5.020268
16	0.073869	1.917399	5.020268
15	4.078477	-1.796995	-2.230929
15	-4.078477	1.796995	2.230929
15	4.065771	-1.796882	-4.487975
15	-4.065771	1.796882	4.487975
49	1.020101	-0.040245	-3.584694
49	-1.020101	0.040245	3.584694
29	-2.006395	1.717416	-1.845708
29	2.006395	-1.717416	1.845708

CuInP2S6 bilayer Aoo [bc'] PBE-D3

E= -93.7006 eV
box: 6.088000 0 0 -3.044000   5.272363 0 0 0 30.000000
Kpoints: 4 4 1
basis: NGX= 36 NGY= 36 NGZ= 168 NGXF= 72 NGYF= 72 NGZF= 336

16	-1.124751	-0.049160	-1.628839
16	1.124751	0.049160	1.628839
16	2.140448	-1.917346	-1.642565
16	-2.140448	1.917346	1.642565
16	-3.950157	1.851215	-1.669002
16	3.950157	-1.851215	1.669002
16	2.966910	-0.171381	-5.044255
16	-2.966910	0.171381	5.044255
16	0.196245	1.708912	-5.043485
16	-0.196245	-1.708912	5.043485
16	-0.046002	-1.630924	-5.046033
16	0.046002	1.630924	5.046033
15	-1.998353	-1.792016	-2.172402
15	1.998353	1.792016	2.172402
15	-2.004919	-1.788027	-4.428315
15	2.004919	1.788027	4.428315
49	1.037153	-0.039836	-3.077044
49	-1.037153	0.039836	3.077044
29	-2.004807	1.725478	-4.708654
29	2.004807	-1.725478	4.708654

CuInP2S6 trilayer AiiF [b'c'd] PBE-D3

E= -141.0876 eV
box: 6.088000 0 0 -3.044000   5.272363 0 0 0 43.000000
Kpoints: 4 4 1
basis: NGX= 36 NGY= 36 NGZ= 250 NGXF= 72 NGYF= 72 NGZF= 500

16	-0.892758	0.023336	-11.640577
16	1.923816	1.896758	-11.652852
16	1.936200	-1.872050	-11.647467
16	3.868473	-1.711578	-8.259942
16	1.115699	0.170513	-8.246853
16	-1.968349	1.613238	-8.237126
16	-0.082567	-1.610044	-5.049016
16	2.919195	-0.168125	-5.037009
16	0.166783	1.712949	-5.022839

```
 16   -3.960802     1.878700    -1.662699
 16    2.125918    -1.898906    -1.631550
 16   -1.151799    -0.016648    -1.653435
 16    0.893481     0.174815     1.581928
 16   -2.111281     1.616839     1.594427
 16   -1.861866    -1.709319     1.615076
 16    0.063045    -1.870643     4.996239
 16    2.894861     0.024604     4.994766
 16    0.076806     1.896793     5.001462
 15   -0.024729     1.775380   -11.120379
 15   -0.011906     1.779272    -8.864334
 15    4.048651    -1.777387    -4.426048
 15   -2.013336    -1.771780    -2.171900
 15   -4.068240     1.779171     2.214340
 15   -4.062391     1.774559     4.470050
 49    3.015987     0.019867   -10.218047
 49    1.025900    -0.018727    -3.063391
 49   -1.015563     0.023207     3.566328
 29   -0.008323    -1.733209    -8.478414
 29   -2.044499     1.735255    -4.802338
 29    2.013980    -1.728259     1.837890
```

```
CuInP2S6 trilayer AiiAoo [bc'd] PBE-D3

E= -141.0000 eV
box:   6.088000 0 0  -3.044000   5.272363 0 0 0  43.000000
Kpoints:  4  4  1
basis: NGX=  36 NGY=  36 NGZ= 250 NGXF=  72 NGYF=  72 NGZF= 500

 16   -0.900788     0.030978   -11.615550
 16    1.916931     1.902289   -11.623859
 16    1.927529    -1.865643   -11.611021
 16    3.866113    -1.708899    -8.231804
 16    1.114268     0.173036    -8.218475
 16   -1.969701     1.614221    -8.204606
 16   -0.081417    -1.608574    -5.027238
 16    2.920761    -0.167362    -5.011788
 16    0.168838     1.713218    -4.996814
 16   -3.955938     1.868816    -1.652453
 16    2.126236    -1.911548    -1.614241
 16   -1.145418    -0.033158    -1.607444
 16    1.143335     0.038689     1.613683
 16   -2.120020     1.906084     1.628135
 16    3.968775    -1.861563     1.661918
 16   -0.167849    -1.720404     5.024698
 16   -2.940598     0.159796     5.029227
 16    0.073923     1.619501     5.035034
 15   -0.030785     1.781167   -11.089545
 15   -0.014212     1.781622    -8.833714
 15    4.050051    -1.776118    -4.401142
 15   -2.014210    -1.779343    -2.146963
 15   -4.069197     1.780611     2.158318
 15   -4.057047     1.776553     4.413368
 49    3.013729     0.026820   -10.186808
 49    1.022516    -0.029786    -3.038731
 49   -1.015834     0.030513     3.065159
 29   -0.009959    -1.731648    -8.448588
 29   -2.042601     1.736370    -4.771460
 29    2.032952    -1.737659     4.688992
```

```
CuInP2S6 trilayer FF [bcd]

E= -140.9938 eV
box:   6.088000 0 0  -3.044000   5.272363 0 0 0  43.000000
Kpoints:  4  4  1
basis: NGX=  36 NGY=  36 NGZ= 250 NGXF=  72 NGYF=  72 NGZF= 500
```

```
 16   -0.891347    0.025753   -11.605956
 16    1.926895    1.895754   -11.614372
 16    1.941794   -1.869140   -11.608703
 16    3.869228   -1.707888    -8.227198
 16    1.113514    0.176721    -8.193413
 16   -1.970984    1.617509    -8.205725
 16   -0.093865   -1.899179    -4.983895
 16   -2.905094   -0.017027    -4.962266
 16   -0.094813    1.877607    -4.948678
 16    1.863488    1.710638    -1.597745
 16    2.109546   -1.612254    -1.571061
 16   -0.891930   -0.172458    -1.561943
 16    1.150708    0.024729     1.662915
 16   -2.120593    1.897934     1.648619
 16    3.972762   -1.868398     1.673180
 16   -0.171268   -1.716925     5.054536
 16   -2.943175    0.163684     5.056604
 16    0.069349    1.623973     5.056615
 15   -0.021722    1.775698   -11.081254
 15   -0.013998    1.780748    -8.825056
 15    4.045547   -1.771065    -4.442426
 15    4.066996   -1.777272    -2.187830
 15    2.017217    1.776404     2.186284
 15   -4.059898    1.780700     4.441273
 49    3.020188    0.023228   -10.175983
 49    1.007232   -0.023771    -3.547790
 49   -1.019800    0.021434     3.091207
 29   -0.006866   -1.726570    -8.446066
 29   -2.011980    1.730882    -1.815427
 29    2.029254   -1.732870     4.717845
```

```
CuInP2S6 quadlayer AiiFF [a'b'c'd] PBE-D3

E= -188.3009 eV
box:   6.088000 0 0  -3.044000   5.272363 0 0 0  43.000000
Kpoints:  4  4  1
basis: NGX=  36 NGY=  36 NGZ= 250 NGXF=  72 NGYF=  72 NGZF= 500

 16   -0.894549    0.024666   -11.632272
 16    1.921048    1.898613   -11.643560
 16    1.932412   -1.871216   -11.635863
 16    3.867268   -1.711662    -8.250593
 16    1.114552    0.169764    -8.238118
 16   -1.969735    1.612736    -8.227129
 16   -0.083711   -1.612308    -5.043504
 16    2.918801   -0.170248    -5.030645
 16    0.165843    1.711050    -5.016806
 16   -3.961461    1.875154    -1.658626
 16    2.125193   -1.901669    -1.625873
 16   -1.152204   -0.019579    -1.647968
 16    0.887439    0.171365     1.570440
 16   -2.115698    1.612782     1.581037
 16   -1.869767   -1.711879     1.606076
 16    0.085662   -1.878641     4.959018
 16    2.896165    0.015855     4.969504
 16    0.084074    1.899691     4.988470
 16    1.923186   -1.614421     8.207794
 16   -1.161951   -0.173856     8.190683
 16   -3.917210    1.711977     8.226062
 16   -1.993381    1.874093    11.608013
 16    4.108950   -1.891350    11.612143
 16    0.838979   -0.020912    11.605149
 15   -0.027219    1.776531   -11.110147
 15   -0.013166    1.778961    -8.854271
 15    4.047943   -1.779604    -4.420098
 15   -2.013887   -1.774693    -2.166444
 15    2.014683    1.776611     2.197448
```

```
 15   -4.054206    1.770144    4.451140
 15   -0.034981   -1.776633    8.824635
 15   -0.030078   -1.770859   11.079999
 49    3.014771    0.021282  -10.211013
 49    1.024716   -0.021995   -3.059875
 49   -1.014253    0.021485    3.554647
 49    3.016383   -0.017873   10.175346
 29   -0.009659   -1.733917   -8.471292
 29   -2.045152    1.733372   -4.796820
 29    2.007105   -1.731847    1.828402
 29   -0.041691    1.729031    8.445149
```

```
CuInP2S6 quadlayer AiiFAoo [abc'd] PBE-D3

E= -188.2130 eV
box:   6.088000 0 0  -3.044000   5.272363 0 0 0  43.000000
Kpoints:  4  4  1
basis: NGX=  36 NGY=  36 NGZ= 250 NGXF=  72 NGYF=  72 NGZF= 500

 16   -0.895190    0.028678  -11.618687
 16    1.922592    1.899305  -11.629004
 16    1.936738   -1.866805  -11.624363
 16    3.866911   -1.708197   -8.235654
 16    1.114322    0.173960   -8.223264
 16   -1.969768    1.616705   -8.213069
 16   -0.080964   -1.606266   -5.027764
 16    2.922083   -0.163391   -5.012147
 16    0.168535    1.716989   -4.998353
 16   -3.957085    1.872171   -1.651520
 16    2.125705   -1.908456   -1.615371
 16   -1.146135   -0.030182   -1.607674
 16    1.145855    0.027093    1.626610
 16   -2.124620    1.903433    1.636713
 16    3.962403   -1.873504    1.669154
 16   -0.173764   -1.716967    5.014630
 16   -2.930748    0.166285    5.047924
 16    0.072953    1.607260    5.042258
 16    1.907950   -1.901409    8.274214
 16   -0.909097   -0.029422    8.286484
 16    1.915529    1.864488    8.297978
 16   -2.233722    1.715957   11.683725
 16   -1.991295   -1.623043   11.683484
 16    1.084459   -0.163370   11.681892
 15   -0.026026    1.779632  -11.095920
 15   -0.013384    1.782664   -8.839734
 15    4.050387   -1.772863   -4.401189
 15   -2.014572   -1.776293   -2.146779
 15    2.015140    1.771972    2.167834
 15   -4.058188    1.771135    4.422682
 15   -0.041828   -1.779738    8.812841
 15   -0.032280   -1.781670   11.068366
 49    3.016204    0.023476  -10.191356
 49    1.022452   -0.025582   -3.038959
 49   -1.021999    0.025262    3.064676
 49    3.008196   -0.026347    9.715411
 29   -0.009882   -1.729656   -8.452884
 29   -2.041998    1.739821   -4.772030
 29    2.037886   -1.736897    4.786955
 29   -0.032535    1.733773   11.352128
```

```
CuInP2S6 quadlayer AiiAooAii [a'bc'd] PBE-D3

E= -188.3072 eV
box:   6.088000 0 0  -3.044000   5.272363 0 0 0  43.000000
Kpoints:  4  4  1
basis: NGX=  36 NGY=  36 NGZ= 250 NGXF=  72 NGYF=  72 NGZF= 500
```

```
 16   -0.898789    0.028778  -11.613479
 16    1.918358    1.901028  -11.624112
 16    1.931218   -1.866440  -11.616334
 16    3.865091   -1.708330   -8.232423
 16    1.112713    0.173107   -8.220194
 16   -1.971027    1.615249   -8.206237
 16   -0.080977   -1.608733   -5.026552
 16    2.921059   -0.166630   -5.011454
 16    0.168690    1.713500   -4.996804
 16   -3.956446    1.868937   -1.653849
 16    2.125006   -1.912496   -1.616609
 16   -1.146744   -0.033200   -1.607595
 16    1.144263    0.032559    1.606031
 16   -2.127495    1.912570    1.615597
 16    3.954090   -1.869661    1.650851
 16   -0.173390   -1.713585    4.995285
 16   -2.925584    0.166523    5.009860
 16    0.075974    1.608646    5.023476
 16    1.917779   -1.614835    8.203499
 16   -1.166513   -0.173175    8.216071
 16   -3.918529    1.708212    8.228103
 16   -1.987641    1.867200   11.614057
 16    4.113155   -1.901442   11.618650
 16    0.842152   -0.028080   11.609143
 15   -0.030060    1.780068  -11.091048
 15   -0.015393    1.782286   -8.835337
 15    4.050129   -1.775735   -4.401603
 15   -2.014903   -1.779377   -2.147976
 15    2.012249    1.778807    2.146299
 15   -4.054486    1.775521    4.399913
 15   -0.038415   -1.782220    8.831328
 15   -0.025956   -1.779618   11.086954
 49    3.013187    0.025460  -10.189001
 49    1.021909   -0.029214   -3.039003
 49   -1.025145    0.028255    3.036688
 49    3.020119   -0.023931   10.185708
 29   -0.011501   -1.731344   -8.449116
 29   -2.042165    1.736524   -4.772307
 29    2.037685   -1.736490    4.769987
 29   -0.042450    1.731306    8.443773
```

```
CuInP2S6 quadlayer FFF [abcd] PBE-D3

E= -188.2114 eV
box:   6.088000 0 0  -3.044000   5.272363 0 0 0  43.000000
Kpoints:  4  4  1
basis: NGX=  36 NGY=  36 NGZ= 250 NGXF=  72 NGYF=  72 NGZF= 500

 16   -0.897466    0.030191  -11.613301
 16    1.923011    1.896342  -11.616939
 16    1.936573   -1.867151  -11.604682
 16    3.867336   -1.709891   -8.228040
 16    1.111678    0.175237   -8.194533
 16   -1.972371    1.615944   -8.206444
 16   -0.093020   -1.897989   -4.978058
 16   -2.905176   -0.019907   -4.955109
 16   -0.090642    1.873615   -4.942429
 16    1.864496    1.708317   -1.591440
 16    2.112633   -1.613760   -1.562650
 16   -0.889735   -0.176171   -1.553682
 16    1.147861    0.022569    1.652221
 16   -2.126366    1.899573    1.634996
 16    3.962939   -1.872367    1.669602
 16   -0.170099   -1.710622    5.021019
 16   -2.927574    0.173326    5.054253
 16    0.075536    1.614769    5.045322
 16    1.908536   -1.898845    8.278045
```

```
 16   -0.911264   -0.029319    8.292090
 16    1.916567    1.863579    8.303457
 16   -2.229950    1.715772   11.687404
 16   -1.986845   -1.623824   11.692223
 16    1.086908   -0.164886   11.689277
 15   -0.025063    1.776900  -11.082595
 15   -0.015516    1.778968   -8.826587
 15    4.046662   -1.773121   -4.435163
 15    4.068820   -1.779790   -2.181048
 15    2.012452    1.774168    2.175027
 15   -4.055064    1.778598    4.428659
 15   -0.041672   -1.779324    8.817035
 15   -0.029410   -1.782136   11.073431
 49    3.019281    0.024853  -10.175542
 49    1.007614   -0.026541   -3.536695
 49   -1.024290    0.025117    3.074118
 49    3.008273   -0.026791    9.727357
 29   -0.008669   -1.728056   -8.446584
 29   -2.010186    1.728838   -1.797957
 29    2.041163   -1.729655    4.793321
 29   -0.028746    1.733469   11.360862
```

```
CuInP2S6 bulk F [abcdef] PBE-D3

E=  -94.5681 eV
box:   6.112093   0.000000   0.000000  -3.056047   5.294163   0.000000  -4.061750   0.000000
13.207700
Kpoints:  4  4  2
basis: NGX=  40 NGY=  40 NGZ=  90 NGXF=  64 NGYF=  64 NGZF= 150

 16    2.126468   -1.643534   -1.374853
 16   -2.128861    1.884290    1.819262
 16    1.877166    1.707136   -1.404738
 16   -2.107184   -1.888428    1.854619
 16   -0.901027   -0.194262   -1.365839
 16    1.156059    0.024597    1.839449
 16   -0.097985   -1.909478   -4.784588
 16    0.095593    1.618346    5.229001
 16   -0.076309    1.863240   -4.749231
 16   -0.153708   -1.732324    5.199115
 16    3.186934   -0.049787   -4.764401
 16   -2.931901    0.169074    5.238015
 15    4.085342   -1.803495   -1.989099
 15   -4.080285    1.771771    2.362826
 15    4.062683   -1.796960   -4.241023
 15   -4.057627    1.778306    4.614754
 49    1.014297   -0.041653   -3.327104
 49   -1.016578    0.016462    3.276747
 29   -2.015699    1.719559   -1.588348
 29    2.065519   -1.744747    5.015499
```

```
CuInP2S6 bulk A [a'bc'de'f] PBE-D3

E=  -94.5461 eV
box:   6.107742   0.000000   0.000000  -3.052370   5.294543   0.000000  -4.052272   0.000000
13.226092
Kpoints:  4  4  2
basis: NGX=  40 NGY=  40 NGZ=  90 NGXF=  64 NGYF=  64 NGZF= 150

 16    2.140469   -1.912546   -1.611082
 16   -2.140469    1.912546    1.611082
 16    2.132821    1.875183   -1.656521
 16   -2.132821   -1.875183    1.656521
 16   -1.142031   -0.047006   -1.604302
 16    1.142031    0.047006    1.604302
 16   -0.086065   -1.624439   -5.022430
```

```
 16    0.086065    1.624439    5.022430
 16    0.164050    1.725726   -4.988249
 16   -0.164050   -1.725726    4.988249
 16    2.936244   -0.169414   -5.006119
 16   -2.936244    0.169414    5.006119
 15    4.088096   -1.789417   -2.145976
 15   -4.088096    1.789417    2.145976
 15    4.062185   -1.784846   -4.398587
 15   -4.062185    1.784846    4.398587
 49    1.027079   -0.031351   -3.038976
 49   -1.027079    0.031351    3.038976
 29   -2.051165    1.741080   -4.774968
 29    2.051165   -1.741080    4.774968
```

```
CuBiP2Se6 bilayer F [ab] PBE-D3

E=  -86.2787 eV
box:   6.652400 0 0  -3.326200   5.761147 0 0 0  30.000000
Kpoints:  4  4  1
basis: NGX=  40 NGY=  40 NGZ= 168 NGXF=  80 NGYF=  80 NGZF= 336

 34    0.447977    1.223130   -4.858990
 34   -2.946349    2.656968   -4.858990
 34   -2.490927   -0.999524   -4.858990
 34   -0.547890   -0.847758   -1.271352
 34   -0.654975    2.829965   -1.271352
 34   -3.786434    0.898366   -1.271352
 34    3.775525   -1.262767    1.883985
 34    0.344850   -2.638316    1.883985
 34    0.868927    1.020509    1.883985
 34   -0.467534   -0.886635    5.446034
 34    2.664716   -2.842153    5.446034
 34    2.792120    0.848215    5.446034
 15   -1.663099    0.960191   -4.286659
 15   -1.663099    0.960191   -2.003216
 15    1.663099   -0.960191    2.445639
 15    1.663099   -0.960191    4.731941
 29   -4.989299    2.880573    4.901089
 29   -4.989299    2.880573   -1.777899
 83    1.663099   -0.960191   -3.340719
 83   -1.663099    0.960191    3.359208
```

```
CuBiP2Se6 bilayer Aii [b'c] PBE-D3

E=  -86.3645 eV
box:   6.652400 0 0  -3.326200   5.761147 0 0 0  30.000000
Kpoints:  4  4  1
basis: NGX=  40 NGY=  40 NGZ= 168 NGXF=  80 NGYF=  80 NGZF= 336

 34    2.508701    0.044471   -5.159351
 34   -2.508701   -0.044471    5.159351
 34   -1.292864    2.150363   -5.159351
 34    1.292864   -2.150363    5.159351
 34   -1.215838   -2.194833   -5.159351
 34    1.215838    2.194833    5.159351
 34    1.203469   -1.835380   -1.569796
 34   -1.203469    1.835380    1.569796
 34    0.987751    1.959925   -1.569796
 34   -0.987751   -1.959925    1.569796
 34   -2.191221   -0.124545   -1.569796
 34    2.191221    0.124545    1.569796
 15    3.326200   -1.920382   -4.589976
 15   -3.326200    1.920382    4.589976
 15    3.326200   -1.920382   -2.304807
 15   -3.326200    1.920382    2.304807
 29   -3.326200    1.920382   -2.013733
```

```
 29    3.326200   -1.920382    2.013733
 83    0.000000    0.000000   -3.661162
 83    0.000000    0.000000    3.661162
```

```
CuBiP2Se6 bilayer Aoo [ab'] PBE-D3

E=  -86.2222 eV
box:   6.652400 0 0  -3.326200   5.761147 0 0 0  30.000000
Kpoints:  4  4  1
basis: NGX=  40 NGY=  40 NGZ= 168 NGXF=  80 NGYF=  80 NGZF= 336

 34   -2.785123   -0.849964   -5.178760
 34    2.785123    0.849964    5.178760
 34    0.465552    0.893569   -5.178760
 34   -0.465552   -0.893569    5.178760
 34   -2.669730    2.836970   -5.178760
 34    2.669730   -2.836970    5.178760
 34   -3.772593    1.235963   -1.600460
 34    3.772593   -1.235963    1.600460
 34   -0.847179   -1.004570   -1.600460
 34    0.847179    1.004570    1.600460
 34   -0.369528    2.649180   -1.600460
 34    0.369528   -2.649180    1.600460
 15   -1.663100    0.960191   -4.463831
 15    1.663100   -0.960191    4.463831
 15   -1.663100    0.960191   -2.177407
 15    1.663100   -0.960191    2.177407
 29    1.663100    2.880573   -4.621532
 29   -1.663100   -2.880573    4.621532
 83    1.663100   -0.960191   -3.097981
 83   -1.663100    0.960191    3.097981
```

```
CuBiP2Se6 bilayer F' [bc] PBE-D3

E=  -86.2749 eV
box:   6.652400 0 0  -3.326200   5.761147 0 0 0  30.000000
Kpoints:  4  4  1
basis: NGX=  40 NGY=  40 NGZ= 168 NGXF=  80 NGYF=  80 NGZF= 336

 34    2.518720    0.049125   -4.879391
 34   -2.209073    0.107354    5.470828
 34   -2.185886   -0.118305   -1.304489
 34    2.482981   -0.024092    1.872624
 34    1.195398   -1.833881   -1.304489
 34   -1.220626    2.162371    1.872624
 34    0.990488    1.952185   -1.304489
 34   -1.262355   -2.138279    1.872624
 34   -1.301904    2.156713   -4.879391
 34    1.011565   -1.966790    5.470828
 34   -1.216816   -2.205838   -4.879391
 34    1.197508    1.859436    5.470828
 15    3.326200   -1.920382   -2.026595
 15   -3.326200    1.920382    2.474104
 15    3.326200   -1.920382   -4.311490
 15   -3.326200    1.920382    4.759561
 29   -3.326200    1.920382   -1.764215
 29    3.326200   -1.920382    4.913651
 83    0.000000    0.000000   -3.394360
 83    0.000000    0.000000    3.394360
```

```
CuBiP2Se6 bilayer A'ii [a'b] PBE-D3

E=  -86.3094 eV
box:   6.652400 0 0  -3.326200   5.761147 0 0 0  30.000000
```

```
Kpoints:  4  4  1
basis: NGX=  40 NGY=  40 NGZ= 168 NGXF=  80 NGYF=  80 NGZF= 336

 34   -2.493410   -0.996963   -5.170846
 34    2.493410    0.996963    5.170846
 34    0.447000    1.219699   -5.170846
 34   -0.447000   -1.219699    5.170846
 34   -2.942891    2.657838   -5.170846
 34    2.942891   -2.657838    5.170846
 34   -3.789810    0.905784   -1.587121
 34    3.789810   -0.905784    1.587121
 34   -0.552627   -0.854391   -1.587121
 34    0.552627    0.854391    1.587121
 34   -0.646863    2.829180   -1.587121
 34    0.646863   -2.829180    1.587121
 15   -1.663100    0.960191   -4.595489
 15    1.663100   -0.960191    4.595489
 15   -1.663100    0.960191   -2.309846
 15    1.663100   -0.960191    2.309846
 29    1.663100    2.880573   -2.109608
 29   -1.663100   -2.880573    2.109608
 83    1.663100   -0.960191   -3.652206
 83   -1.663100    0.960191    3.652206
```

```
CuBiP2Se6 bilayer A'oo [bc'] PBE-D3

E=  -86.1724 eV
box:   6.652400 0 0  -3.326200   5.761147 0 0 0  30.000000
Kpoints:  4  4  1
basis: NGX=  40 NGY=  40 NGZ= 168 NGXF=  80 NGYF=  80 NGZF= 336

 34    2.212377   -0.107423   -5.238201
 34   -2.212377    0.107423    5.238201
 34   -1.013158    1.969686   -5.238201
 34    1.013158   -1.969686    5.238201
 34   -1.199220   -1.862262   -5.238201
 34    1.199220    1.862262    5.238201
 34    1.216824   -2.161052   -1.643426
 34   -1.216824    2.161052    1.643426
 34    1.263114    2.134327   -1.643426
 34   -1.263114   -2.134327    1.643426
 34   -2.479939    0.026725   -1.643426
 34    2.479939   -0.026725    1.643426
 15    3.326200   -1.920382   -4.522082
 15   -3.326200    1.920382    4.522082
 15    3.326200   -1.920382   -2.235508
 15   -3.326200    1.920382    2.235508
 29   -3.326200    1.920382   -4.675901
 29    3.326200   -1.920382    4.675901
 83    0.000000    0.000000   -3.169092
 83    0.000000    0.000000    3.169092
```

```
CuBiP2Se6 bulk F [abcdef] PBE-D3

E= -261.3515 eV
box:   6.583651   0.000000   0.000000  -3.291825   5.701609   0.000000   0.000000   0.000000
40.425613
Kpoints:  4  4  1
basis: NGX=  48 NGY=  48 NGZ= 300 NGXF=  70 NGYF=  70 NGZF= 432

 34    2.542701    0.101291   -4.857137
 34   -2.166002    0.111663    5.476995
 34   -1.359071    2.151398   -4.857137
 34    0.986298   -1.931645    5.476995
 34   -1.183630   -2.252689   -4.857137
 34    1.179704    1.819982    5.476995
```

```
 34    0.986298    1.869428   -7.998208
 34   -1.359073   -1.649675    8.618068
 34   -0.749124   -1.799244  -18.332340
 34    1.125824    2.012200   18.952201
 34   -2.112121   -0.080555   -7.998208
 34    2.108197   -0.352154    8.618068
 34    1.932753    0.250861  -18.332340
 34   -2.305529   -0.031107   18.952201
 34    1.125823   -1.788873   -7.998208
 34   -0.749124    2.001829    8.618068
 34   -1.183629    1.548383  -18.332340
 34    1.179704   -1.981093   18.952201
 34   -2.134692   -0.119508   -1.292084
 34    2.483254   -0.061390    1.869876
 34    1.170843   -1.788943   -1.292084
 34   -1.188462    2.181257    1.869876
 34    0.963849    1.908452   -1.292084
 34   -1.294793   -2.119866    1.869876
 34    2.103362    0.280721  -11.605326
 34   -2.120982    0.111593   12.183118
 34    1.157133   -2.020044  -14.767292
 34   -0.808571    1.839147   15.345080
 34   -1.294792    1.681205  -11.605326
 34    0.963849   -1.892620   12.183118
 34    1.170843    2.012129  -14.767292
 34   -1.188462   -1.619816   15.345080
 34   -0.808570   -1.961925  -11.605326
 34    1.157133    1.781028   12.183118
 34   -2.327976    0.007915  -14.767292
 34    1.997033   -0.219330   15.345080
 15    3.291825   -1.900536   -4.317276
 15   -3.291825    1.900536    4.737455
 15    0.000000    0.000000   -8.737746
 15    0.000000    0.000000    9.157928
 15   -3.291825    1.900536  -17.792481
 15    3.291825   -1.900536   18.212660
 15    3.291825   -1.900536   -2.030434
 15   -3.291825    1.900536    2.454176
 15    0.000000    0.000000  -11.021029
 15    0.000000    0.000000   11.444773
 15   -3.291825    1.900536  -15.505637
 15    3.291825   -1.900536   15.929380
 29   -3.291825    1.900536   -1.764998
 29    3.291825   -1.900536    4.956550
 29   -3.291825    1.900536   -8.518657
 29    3.291825   -1.900536   11.710206
 29    0.000000    0.000000  -15.240201
 29    0.000000    0.000000   18.431753
 83    0.000000    0.000000   -3.397430
 83    0.000000    0.000000    3.397430
 83    3.291825   -1.900536  -10.077772
 83   -3.291825    1.900536   10.077774
 83    3.291825   -1.900536  -16.872633
 83   -3.291825    1.900536   16.872636
```

```
CuBiP2Se6 bulk A [ab'cd'ef'] PBE-D3

E= -261.3940 eV
box:   6.590730   0.000000   0.000000  -3.295365   5.707740   0.000000   0.000000   0.000000
40.306381
Kpoints:  4  4  1
basis: NGX=  48 NGY=  48 NGZ= 294 NGXF=  70 NGYF=  70 NGZF= 432

 34    2.512563    0.075938   -5.126141
 34   -2.512563   -0.075938    5.126141
 34   -1.322046    2.137974   -5.126141
 34    1.322046   -2.137974    5.126141
 34   -1.190517   -2.213913   -5.126141
```

34	1.190517	2.213913	5.126141
34	1.322046	1.667186	-8.309318
34	-1.322046	-1.667186	8.309318
34	-0.782802	-1.826642	-18.561601
34	0.782802	1.826642	18.561601
34	-2.104848	0.311333	-8.309318
34	2.104848	-0.311333	8.309318
34	1.973320	0.235394	-18.561601
34	-1.973320	-0.235394	18.561601
34	0.782802	-1.978518	-8.309318
34	-0.782802	1.978518	8.309318
34	-1.190517	1.591248	-18.561601
34	1.190517	-1.591248	18.561601
34	-2.152191	-0.119418	-1.542375
34	2.152191	0.119418	1.542375
34	1.179514	-1.804143	-1.542375
34	-1.179514	1.804143	1.542375
34	0.972677	1.923561	-1.542375
34	-0.972677	-1.923561	1.542375
34	-0.972678	1.881601	-11.893084
34	0.972678	-1.881601	11.893084
34	1.143175	-2.021996	-14.977836
34	-1.143175	2.021996	14.977836
34	-1.143175	-1.783164	-11.893084
34	1.143175	1.783164	11.893084
34	1.179513	2.001017	-14.977836
34	-1.179513	-2.001017	14.977836
34	2.115853	-0.098437	-11.893084
34	-2.115853	0.098437	11.893084
34	-2.322688	0.020980	-14.977836
34	2.322688	-0.020980	14.977836
15	3.295365	-1.902580	-4.559742
15	-3.295365	1.902580	4.559742
15	0.000000	0.000000	-8.875716
15	0.000000	0.000000	8.875716
15	-3.295365	1.902580	-17.995204
15	3.295365	-1.902580	17.995204
15	3.295365	-1.902580	-2.276098
15	-3.295365	1.902580	2.276098
15	0.000000	0.000000	-11.159364
15	0.000000	0.000000	11.159364
15	-3.295365	1.902580	-15.711557
15	3.295365	-1.902580	15.711557
29	-3.295365	1.902580	-2.020202
29	3.295365	-1.902580	2.020202
29	-3.295365	1.902580	-11.415259
29	3.295365	-1.902580	11.415259
29	0.000000	0.000000	-15.455664
29	0.000000	0.000000	15.455664
83	0.000000	0.000000	-3.657534
83	0.000000	0.000000	3.657534
83	3.295365	-1.902580	-9.777924
83	-3.295365	1.902580	9.777924
83	3.295365	-1.902580	-17.092996
83	-3.295365	1.902580	17.092996
